# Marked effect of topical application of Chinese medicine combined with moxibustion in a case of refractory malignant ascites in diffuse liver cancer

**DOI:** 10.3389/fmed.2026.1751741

**Published:** 2026-03-04

**Authors:** Dou-dou Feng, Xing-ping Zhang, Lei Guo, Chuan He

**Affiliations:** 1 Affiliated Hospital of Traditional Chinese Medicine of Xinjiang Medical University, Urumqi, Xinjiang, China; 2 Fourth Clinical Medical College of Xinjiang Medical University, Urumqi, Xinjiang, China; 3 Department of Oncology, The People’s Hospital of Laibin Xingbin District, Laibin, Guangxi, China

**Keywords:** moxibustion, topical application of Chinese medicine, Shenque acupoint (CV8), complications of cancer, refractory malignant ascites, portal vein tumor thrombosis, diffuse liver cancer

## Abstract

**Background:**

Although diffuse hepatocellular carcinoma (HCC) is rarely encountered in clinical practice, it frequently leads to the refractory complication of malignant ascites. Conventional treatments such as diuretics, therapeutic paracentesis, and intraperitoneal chemotherapy often yield suboptimal outcomes.

**Case presentation:**

We report a detailed case of a 55-year-old female patient with diffuse HCC (CNLC stage IV, Child-Pugh C), portal vein tumor thrombus, and malignant ascites refractory to diuretics. Following confirmation of the diagnosis, she was treated with a novel approach: topical application of a specific herbal paste (containing Asarum sieboldii, *Cinnamomum cassia*, Zanthoxylum bungeanum, Astragalus membranaceus, and *Solanum nigrum*) at the Shenque acupoint (CV8) combined with daily one-hour moxibustion. After 1 month of treatment, her abdominal circumference decreased markedly from 86 cm to 71 cm, and her ECOG performance status improved from 3 to 2. The treatment was well-tolerated with no observed adverse effects.

**Clinical context:**

To provide broader clinical context, we briefly note the outcomes of three additional patients with advanced HCC and malignant ascites managed during the same period: two who received the same herbal paste with TDP irradiation (without moxibustion) showed stabilization of ascites, while one untreated patient progressed rapidly.

**Conclusion:**

This detailed case report demonstrates that topical traditional Chinese medicine (TCM) therapy combined with moxibustion at the Shenque acupoint (CV8) was associated with a marked reduction in refractory malignant ascites in one patient with advanced diffuse hepatocellular carcinoma. The treatment was well-tolerated, with no observed adverse effects, and the patient reported subjective symptom relief and voluntarily continued therapy at home, suggesting good acceptability. This unique, non-invasive, acupoint-specific combinatorial strategy introduces a novel approach worthy of further investigation. While these preliminary findings are hypothesis-generating and supported by contextual observations from three additional patients, they require validation through larger, controlled studies to establish efficacy, safety, and underlying mechanisms.

## Introduction

Primary liver cancer, of which hepatocellular carcinoma (HCC) is the most common histologic type, represents a significant global health burden. It is the sixth most frequently diagnosed cancer and the third leading cause of cancer-related mortality worldwide, with an estimated 905,000 new cases and 830,000 deaths annually (1). The highest incidence rates are observed in East Asia and sub-Saharan Africa, largely attributable to the endemic prevalence of chronic hepatitis B virus infection.

The pathogenesis of HCC is a complex, multistep process typically arising in the context of chronic liver disease and cirrhosis. Key etiological factors include chronic viral hepatitis (HBV and HCV), alcohol-related liver disease, and increasingly, metabolic dysfunction-associated steatotic liver disease (MASLD). These insults lead to cycles of hepatocyte injury, inflammation, regeneration, and fibrosis, ultimately resulting in cirrhotic remodeling and the accumulation of genetic and epigenetic alterations that drive malignant transformation (2).

Treatment approaches for HCC are guided by tumor stage, liver function, and performance status, following algorithms such as the Barcelona Clinic Liver Cancer (BCLC) system. Curative-intent options include surgical resection, liver transplantation, and local ablation for early-stage disease. For intermediate-stage HCC, transarterial chemoembolization (TACE) is the standard of care, while advanced-stage disease is managed with systemic therapies, including tyrosine kinase inhibitors (e.g., sorafenib, lenvatinib) and immune checkpoint inhibitors (2). However, these therapies are associated with significant side effects; sorafenib, for instance, frequently causes hand-foot skin reactions, diarrhea, fatigue, and hypertension, often necessitating dose reduction or discontinuation.

Malignant ascites, a common and debilitating complication of advanced HCC, arises from a dual pathophysiology: profound hepatic decompensation with portal hypertension and direct tumor vascular invasion (3, 4). Conventional management includes diuretics, therapeutic paracentesis, and intraperitoneal chemotherapy. However, over 60% of HCC-associated ascites prove refractory to these interventions (3, 5). Repeated paracentesis carries risks of infection, hypovolemia, and protein depletion (6, 7), while providing only temporary relief. This high unmet clinical need has spurred interest in adjunctive and integrative approaches.

Traditional Chinese Medicine (TCM) external therapies have a long history in managing fluid retention disorders. Among these, acupoint stimulation—particularly at the Shenque (CV8) point—has garnered attention for its potential to regulate fluid metabolism. The therapeutic mechanism is theorized to involve neuroimmunological modulation and enhancement of peritoneal absorption (8). The Shenque point, located at the umbilicus, is considered in TCM theory to be a critical site for connecting with the kidneys and spleen, organs governing water metabolism. Prior work by Professor Huang Jinchang documented promising results using moxibustion with a specific herbal paste at CV8 for malignant ascites secondary to gastrointestinal cancers (9). Building upon this foundation, we sought to evaluate this non-invasive approach in the challenging context of diffuse HCC with refractory malignant ascites.

## Case description

A 55-year-old woman was admitted to our hospital with a confirmed diagnosis of diffuse HCC for 4 months, complaining of worsening abdominal distension for 1 week on January 3, 2025. Additional symptoms included fatigue and anorexia. The patient had a history of hepatitis B and was taking antiviral agents regularly. The patient’s illness began in September 2024 with epigastric pain and a palpable mass. Following initial diagnostic workup at outside hospitals that suggested hepatic malignancy, she declined the recommended standard treatment. Her symptoms, including abdominal distension, pain, and fatigue, continued to progress. She presented to our hospital on November 26, 2024. Imaging confirmed a diagnosis of diffuse HCC with malignant ascites. She underwent her first cycle of FOLFOX-HAIC combined with targeted immunotherapy on November 29, 2024. She was hospitalized on December 15, 2024, for treatment-related complications of nausea and vomiting, which resolved with supportive care. By December 19, 2024, the primary symptoms of ascites and abdominal distension began to appear and progressively worsened. No significant family history of liver disease or malignancy was reported.

Physical examination revealed a blood pressure of 128/82 mmHg, a heart rate of 94 bpm, a respiratory rate of 19 breaths/min, and a body temperature of 36.4 °C. Special P. E. examination of the diseased organ system revealed that the abdomen was bulging with shifting dullness positive, abdominal girth: 84.5 cm, the edge of the enlarged liver was palpable 5 cm below the right costal margin, the surface was not smooth, the texture was hard, the liver area experienced percussion pain, the spleen was not palpable under the subcostal margin, there was normal active bowel sounds, and there was no rebound pain in the whole abdomen, which was negative for tenderness, asterixis, or edema of the lower extremities. The patient’s ECOG performance status (PS) was 3.

Following admission, the patient underwent a range of exams and tests, with pertinent test results including the following (Table 1).

**Table 1:** Selected liver function and electrolyte and platelet levels (before vs. after 2 weeks of treatment).

Term	Day 0	Day 15	Unit of measurement	Reference range
TBIL	134.57	115.1	μmol/L	3.4–20.5
D-BIL	73.62	66.08	μmol/L	0–6.8
IB	63.95	47.2	μmol/L	0–14
ALT	55.2	25.2	U/L	7–40
AST	156.8	107.9	U/L	13–35
GGT	58.3	53.3	U/L	7–45
ALP	302.8	219.4	U/L	35–135
K^+^	2.81	3.73	mmol/L	3.5–5.5
AFP	>1,210	>1,210	ng/mL	0–7.4
PLT	72	145	1*10^9^/L	125–350

**Figure 1:**
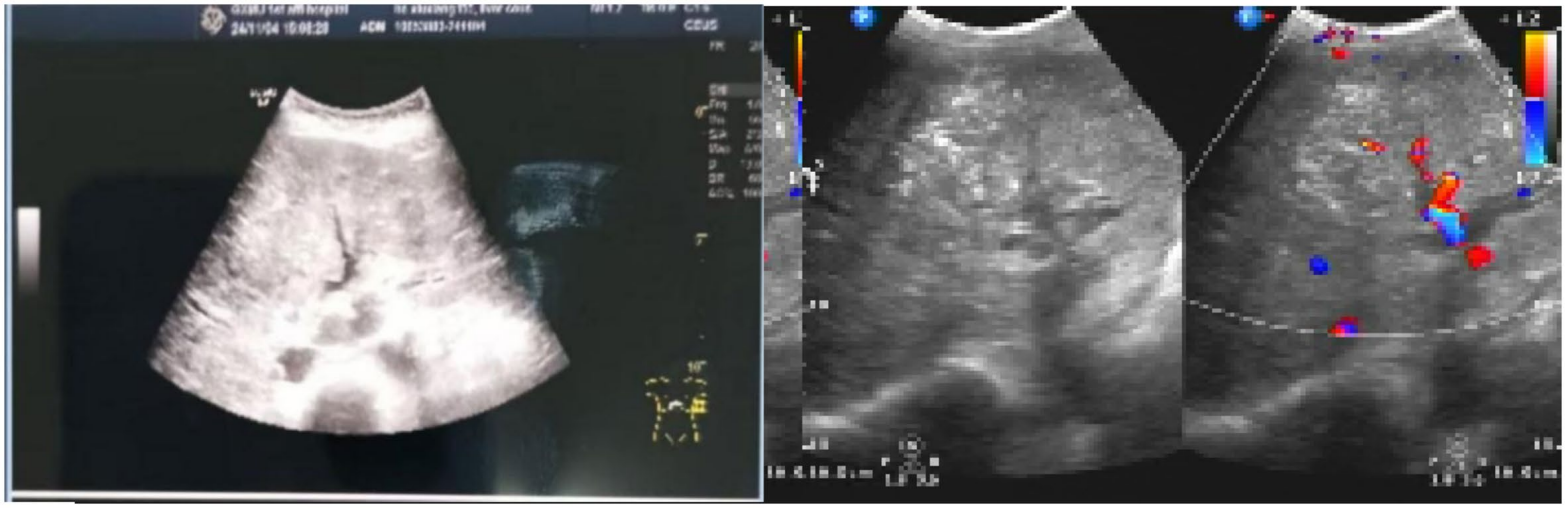
Abdominal ultrasonography before the initiation of the external application of Chinese medicine and moxibustion therapy, showing diffuse liver lesions, right branch embolism of the portal vein, and ascites.

Abdominal ultrasonography before admission revealed diffuse liver lesions with multiple internal solid masses, portal hypertension, left branch embolism of the portal vein (considering Ca and left branch embolism of the portal vein), and ascites (Figure 1).

To ensure diagnostic specificity and minimize confounding influences, we conducted a comprehensive differential diagnosis. The diagnosis of diffuse HCC with malignant ascites was confirmed based on (10): (1) characteristic imaging findings on contrast-enhanced MRI and ultrasound showing diffuse infiltrative lesions with arterial hyperenhancement and washout, (2) markedly elevated serum AFP (>1,210 ng/mL), and (3) the presence of portal vein tumor thrombus on imaging. Other potential causes of ascites were systematically excluded: tuberculous peritonitis (negative interferon-gamma release assay and absence of typical peritoneal findings), heart failure (normal echocardiogram and absence of jugular venous distention), nephrotic syndrome (normal urine protein and renal function), and non-malignant cirrhotic ascites (presence of tumor thrombus and progressive disease despite antiviral therapy). Therefore, the malignant ascites can mainly be attributed solely to advanced HCC with portal hypertension and tumor vascular invasion.

## Chinese medicine combined with moxibustion therapy

Based on the above inspections, the patient was clinically diagnosed with Diffuse HCC classified as China Liver Cancer (CNLC) stage IV or Child–Pugh grade C. The complications included hepatic portal vein thrombus and significant, refractory malignant ascites.

### Therapeutic intervention

Conventional therapies, including diuretics, have proven ineffective. The patient was not considered suitable for intraperitoneal chemotherapy because of their clinical condition. Furthermore, paracentesis was not administered because it does not address the underlying pathophysiological mechanisms responsible for ascites formation, and the condition typically recurs shortly after the procedure (11). Thus, topical application of Chinese medicine combined with moxibustion was used to treat the malignant ascites in this case.

The treatment protocol was derived from Professor Huang Jinchang (*Twenty Years of Oncology Specialized Experience*) (9). The method involved:

Herbal paste preparation: Xixin (Asarum sieboldii) (3 g), Guizhi (*Cinnamomum cassia*) (10 g), Huajiao (Zanthoxylum bungeanum) (10 g), Shenghuangqi (Astragalus membranaceus) (20 g), and Longkui (*Solanum nigrum*) (10 g) were ground into a fine powder.

Application: The powder was mixed with an appropriate medium (e.g., water) to form a paste. This paste was placed directly into the umbilicus (Shenque point, CV8) and around the belly button. In addition, a layer of gauze was placed under the medication for easy cleaning.

Moxibustion: An ignited moxa stick was placed inside a moxibustion box. The box was then positioned over the herbal paste-filled umbilicus.

Procedure duration and frequency: Moxibustion was applied once daily for 60 min per session.

### Follow-up and outcomes

**Figure 2:**
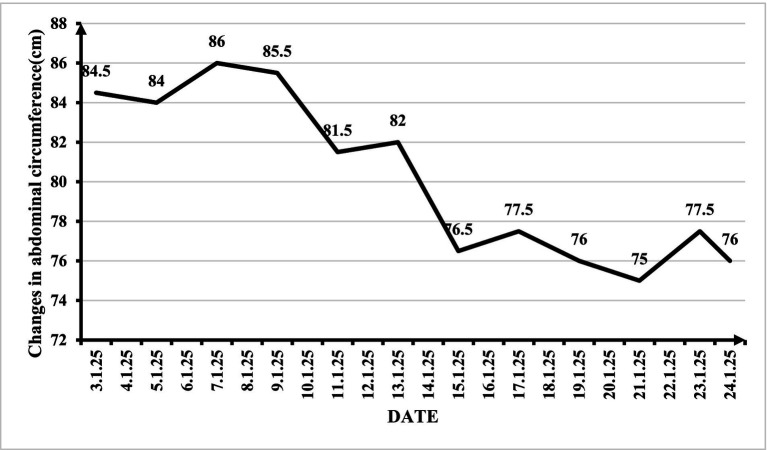
Changes in the patient’s abdominal circumference during the treatment period.

**Figure 3:**
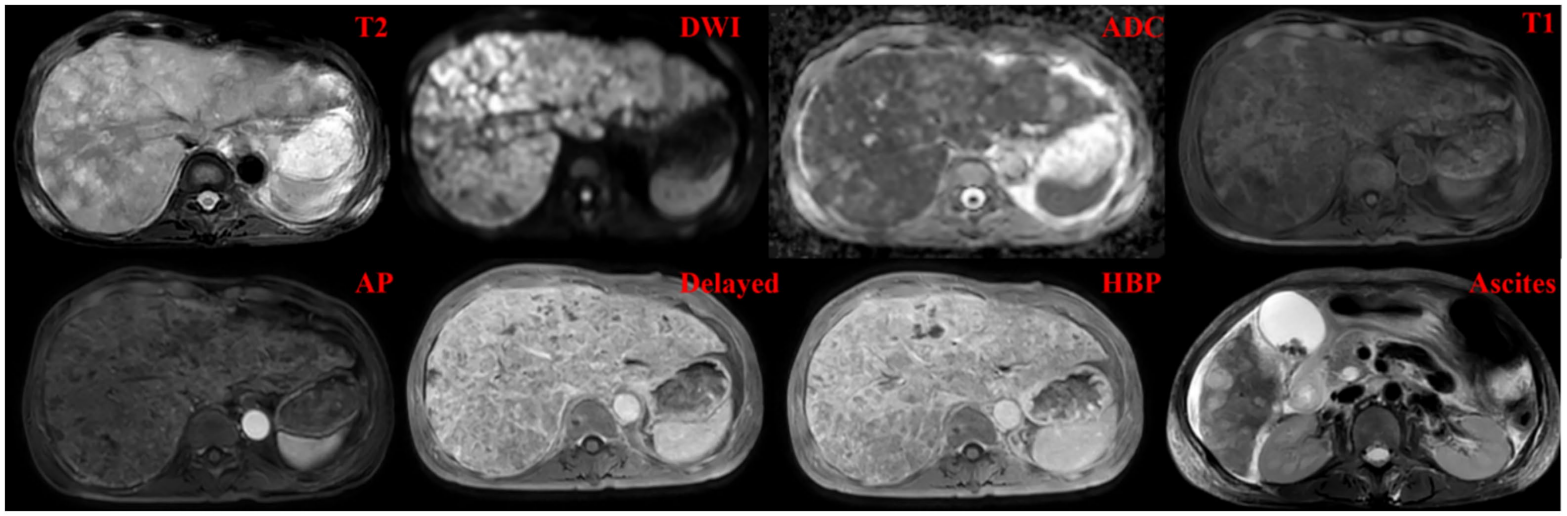
MRI of the liver: T2WI, DWI, apparent diffusion coefficient (ADC), T1WI, contrast-enhanced arterial phase, delayed, and hepatobiliary phases, and ascites after treatment.

The primary outcome measure was the change in abdominal circumference (Figure 2), reflecting the ascites volume. The patient was discharged on January 24, 2025. The pretreatment abdominal circumference was 86 cm. After 1 month of daily therapy, the patient’s abdominal circumference was 71 cm. This represented a significant reduction of 15 cm. The ECOG performance status improved from 3 to 2. Before discharge, the patient underwent an enhanced liver MRI examination (Figure 3), which revealed that the intrahepatic lesions were still enhanced after treatment of diffuse HCC and that there was still a small amount of ascites. From the patient’s perspective, “Abdominal tightness is relieved within a week.” The treatment was well tolerated by the patient, with no reported adverse effects, including local skin burns or systemic reactions.

The patient was discharged with topical Chinese medicine and continued treatment at home; complete resolution of the ascites was reported on February 20.

### Other cases before her (briefly noted)

The report mentioned outcomes in three other patients with advanced HCC, portal vein thrombus, and malignant ascites treated with various types of external therapy:

Patients 2 and 3 (male patients): Treated with the same herbal paste applied to the abdomen. Instead of moxibustion, a TDP (specific electromagnetic wave) lamp was used to irradiate the paste-covered abdomen for 1 h daily. The ascites had stabilized, partial improvement in liver function was observed, and no significant changes in electrolyte levels were noted (Table 2).

**Table 2:** Patient 2: selected liver function and electrolyte levels (before vs. after 1-week of treatment).

Term	Day 0	Day 15	Unit of measurement	Reference range
TBIL	161.55	112.2	μmol/L	3.4–20.5
D-BIL	60.19	61.55	μmol/L	0–6.8
IB	44.92	50.66	μmol/L	0–14
K^+^	4.24	4	mmol/L	3.5–5.5
Na^+^	130.2	131.8	mmol/L	135–146
Cl^-^	93	93.6	mmol/L	95–108
Ca^2+^	1.87	2.05	mmol/L	2.02–2.60
Mg^2+^	0.74	0.71	mmol/L	0.7–11
PO^3-^_4_	0.55	0.57	mmol/L	0.87–1.45

Patient 4: This patient declined the proposed external therapy. His ascites progressed rapidly.

## Discussion

Malignant ascites in advanced HCC poses a significant therapeutic challenge. Conventional methods often provide limited and temporary benefits (3, 4). In traditional Chinese medicine, we often use the Shenque point (CV8) for external treatments. This particular point has been used for centuries to help regulate water metabolism and strengthen the spleen and kidneys. These functions make it especially relevant for treating conditions involving fluid retention, such as ascites, where these TCM concepts come into play (12).

The herbal paste formulation, derived from Professor Huang’s protocol (9), comprises five traditional Chinese medicinals, each with documented bioactive compounds and pharmacological activities relevant to ascites management: Guizhi (*Cinnamomum cassia* Presl): The primary active constituents include cinnamaldehyde, cinnamic acid, and coumarin (13, 14). Cinnamaldehyde has demonstrated anti-inflammatory properties through inhibition of NF-κB and COX-2 pathways, while also exhibiting vasodilatory effects that may enhance local microcirculation (15). Animal studies suggest that cinnamon-derived compounds can improve diuresis and reduce edema formation (16, 17). In traditional Chinese medicine theory, Guizhi is considered to “warm yang and promote circulation,” which aligns with its modern pharmacological profile of enhancing blood flow and reducing fluid retention (13, 14). Xixin (Asarum sieboldii Miq.): Contains volatile oils including methyleugenol, safrole, and asarone, as well as lignans such as asarinin (18). Modern pharmacological studies have identified anti-inflammatory, analgesic, and immunomodulatory activities. Huajiao (Zanthoxylum bungeanum Maxim.): The major bioactive compounds include alkylamides (particularly hydroxy-α-sanshool), lignans, and alkaloids (19). Hydroxy-α-sanshool has been shown to activate TRP channels, potentially influencing visceral sensation and fluid dynamics (20). In TCM, Huajiao is traditionally used to “warm the middle jiao, dispel cold, and alleviate pain,” consistent with its demonstrated effects on sensory neurons and inflammation (19). Shenghuangqi (Astragalus membranaceus (Fisch.) Bunge): Contains astragalosides (saponins), flavonoids (e.g., formononetin), and polysaccharides (21, 22). Astragalus polysaccharides have demonstrated immunomodulatory effects, including enhancement of T-cell function and regulation of cytokine balance (23). Importantly, astragaloside IV has been shown to improve renal function and promote diuresis in experimental models of nephrotic syndrome (24), suggesting a potential mechanism for fluid reduction. Clinical studies have also documented its efficacy in alleviating cancer-related fatigue (21, 22). These pharmacological actions align with its traditional roles in “benefiting water metabolism, reducing edema, and alleviating fatigue” (21, 22). Longkui (*Solanum nigrum* L.): Contains steroidal alkaloids (e.g., solasonine, solamargine), steroidal saponins, and glycoproteins (25–28). Solamargine has demonstrated cytotoxic activity against various cancer cell lines, including HCC, through induction of apoptosis and autophagy (25–28). Additionally, *Solanum nigrum* extracts have shown diuretic and anti-inflammatory properties in preclinical studies (25–28), supporting its traditional use for “clearing heat, removing toxins, promoting diuresis, and reducing swelling”—indications that make it a common TCM herb for ascites and cancer (25–28).

Moxibustion adds warmth and stimulation, potentially enhancing the local microcirculation, absorption, and therapeutic effect of the herbs. Moxibustion can prevent and treat various cancer complications, including bone marrow suppression, fatigue (29), and postoperative lymphedema (30). It has also been shown to improve the quality of life of cancer patients. Moxibustion alone or in combination with chemotherapy can improve the survival rate and inhibit tumor growth in animal models of cancer (31–33). TDP lamp therapy, which is used in other cases, provides thermal and specific electromagnetic stimulation, offering an alternative method to achieve local effects. The observed reduction in abdominal circumference in the index case and stabilization in the two comparison cases suggest a potential local effect on fluid dynamics (34), possibly through enhanced absorption, reduced production, or the modulation of peritoneal permeability. The slight improvement in liver function in TDP patients, while requiring cautious interpretation, is an intriguing observation that warrants further study.

We acknowledge the absence of a control mixture or vehicle control as a significant limitation of this report. In an ideal experimental design, comparing the complete herbal paste against a placebo paste (containing inert ingredients) or against individual herbal components would help isolate the specific contributions of the formulation. However, such controls are challenging to implement in a clinical case report setting, particularly in patients with advanced disease and limited therapeutic options. Moreover, developing an appropriate placebo for topical TCM formulations presents unique challenges related to matching sensory properties (aroma, texture, warmth) that could influence blinding and patient expectations. Future studies should incorporate vehicle-controlled designs with larger sample sizes to validate the specific effects of the herbal formulation. Additionally, preclinical investigations using animal models of malignant ascites could help establish dose–response relationships and elucidate mechanisms of action under controlled conditions.

While the index patient tolerated the treatment well without reported adverse events, it is essential to acknowledge the potential risks associated with both topical herbal application and moxibustion. Cutaneous reactions are the most common concern with topical herbal preparations, including contact dermatitis characterized by erythema, pruritus, vesiculation, or desquamation at the application site (35). The risk is influenced by individual patient sensitivity, specific herbal constituents (particularly those with known sensitizing potential, such as Asarum-derived essential oils), and duration of application. Rarely, systemic absorption through the highly vascularized umbilical region could theoretically lead to systemic effects, although no such reactions were observed in this case. To mitigate these risks, we recommend performing a patch test prior to therapy, limiting initial application duration, ensuring appropriate paste consistency to avoid maceration, and monitoring the application site daily. Moxibustion-related risks include thermal burns or scalding, accidental ignition of bedding, smoke-related respiratory irritation, and discomfort from prolonged positioning (36). In our protocol, these risks were minimized through the use of a moxibustion box that maintains a safe distance from skin, supervision in a well-ventilated room, and patient instruction on immediate reporting of discomfort. For home-based continuation, patients should receive thorough training on safe practices. We emphasize that safety data from this single case cannot be generalized, and systematic adverse event monitoring in larger prospective studies is essential. Patients with diabetes, those on anticoagulant therapy, or those with known herbal allergies should be considered at higher risk and managed with additional precautions.

To contextualize the clinical significance of this finding, we offer the following considerations. First, in the context of refractory malignant ascites secondary to advanced HCC, even modest reductions in fluid accumulation can translate into meaningful symptomatic improvement. The patient reported subjective relief of abdominal tightness within the first week of treatment—a symptom-based outcome that is clinically relevant from a palliative perspective. The subsequent reduction in abdominal circumference from 86 cm to 71 cm represents a decrease of 15 cm, which in clinical practice corresponds to a substantial reduction in intra-abdominal fluid volume. Second, this improvement was achieved without any paracentesis, which is the standard intervention for symptomatic relief in refractory ascites; thus, the patient avoided the risks, discomfort, and temporary nature of repeated drainage procedures. Third, the improvement in ECOG performance status from 3 to 2 indicates enhanced functional capacity, allowing the patient greater independence in daily activities—a clinically meaningful outcome in palliative oncology. Fourth, we acknowledge that statistical significance testing is not applicable to a single case report; the purpose of this report is to describe an observed phenomenon, generate hypotheses, and provide preliminary evidence to support future controlled studies, rather than to establish statistical inference. While we maintain that a 15 cm reduction in abdominal circumference in a patient with refractory malignant ascites is clinically noteworthy, we agree that larger studies with objective volume quantification (e.g., ultrasound or CT volumetry) are needed to confirm the magnitude and consistency of treatment effects.

This case report documents a marked reduction in refractory malignant ascites in a patient with diffuse HCC following topical herbal application combined with moxibustion at Shenque (CV8), with abdominal circumference decreasing from 86 cm to 71 cm and improved performance status. Despite these encouraging findings, the small sample size, lack of controls, absence of objective ascites quantification, and speculative mechanisms preclude definitive conclusions. These hypothesis-generating observations warrant rigorous evaluation in future prospective trials with larger samples, appropriate controls, blinded assessment, and objective outcome measures to validate efficacy, establish safety, and elucidate mechanisms of action.

## Conclusion

This detailed case report, supplemented by observational context from three additional patients, suggests that external TCM therapy involving moxibustion over a specific herbal paste applied at Shenque point (CV8), as per Professor Huang’s protocol, may be a beneficial adjunctive or alternative approach for managing malignant ascites in patients with advanced HCC. The index patient demonstrated a marked reduction in ascites volume (abdominal circumference decreasing from 86 cm to 71 cm) and improved performance status, with good tolerability. The three additional patients are presented only to provide clinical context; two showed stabilization with a related approach (herbal paste with TDP irradiation), while one untreated patient progressed, and do not constitute formal comparative evidence. While these collective observations are encouraging, they are preliminary and hypothesis-generating. The core evidence of this report remains the detailed single case, and rigorous prospective studies with larger sample sizes, appropriate controls, and objective ascites quantification are essential to validate efficacy and establish the therapeutic role of this non-invasive modality.

## Data Availability

The original contributions presented in the study are included in the article/Supplementary material, further inquiries can be directed to the corresponding author.
